# Targeting Mitochondrial Function to Treat Quiescent Tumor Cells in Solid Tumors

**DOI:** 10.3390/ijms161126020

**Published:** 2015-11-13

**Authors:** Xiaonan Zhang, Angelo de Milito, Maria Hägg Olofsson, Joachim Gullbo, Padraig D’Arcy, Stig Linder

**Affiliations:** 1 Department of Medical and Health Sciences, Linköping University, SE-581 83 Linköping, Sweden; padraig.darcy@liu.se; 2 Department of Oncology-Pathology, Karolinska Institute, SE-171 76 Stockholm, Sweden; Angelo.De-Milito@ki.se (A.M.); Maria.Hagg@ki.se (M.H.O.); 3 Department of Immunology, Genetics and Pathology, Section of Oncology, Uppsala University, 751 85 Uppsala, Sweden; joachim.gullbo@medsci.uu.se

**Keywords:** cancer therapy, solid tumor, mitochondria, oxidative phosphorylation, glucose, multicellular tumor spheroids

## Abstract

The disorganized nature of tumor vasculature results in the generation of microenvironments characterized by nutrient starvation, hypoxia and accumulation of acidic metabolites. Tumor cell populations in such areas are often slowly proliferating and thus refractory to chemotherapeutical drugs that are dependent on an active cell cycle. There is an urgent need for alternative therapeutic interventions that circumvent growth dependency. The screening of drug libraries using multicellular tumor spheroids (MCTS) or glucose-starved tumor cells has led to the identification of several compounds with promising therapeutic potential and that display activity on quiescent tumor cells. Interestingly, a common theme of these drug screens is the recurrent identification of agents that affect mitochondrial function. Such data suggest that, contrary to the classical Warburg view, tumor cells in nutritionally-compromised microenvironments are dependent on mitochondrial function for energy metabolism and survival. These findings suggest that mitochondria may represent an “Achilles heel” for the survival of slowly-proliferating tumor cells and suggest strategies for the development of therapy to target these cell populations.

## 1. Solid Tumors Contain Cell Populations with Limited Sensitivity to Treatment

Cancer drug discovery is focused on the development of agents that selectively inhibit the growth of cancer cells while maintaining a therapeutic window towards normal cells. While a relatively simple idea, this strategy is confounded by the fact that cancer cells within a tumor mass are not uniform entities, but rather a heterogeneous cell population with different growth properties and thus drug sensitivities. Non-proliferative or quiescent cells in particular are notoriously difficult to treat and represent the greatest hurdle to the elimination of cancer in patients [1,2]. While this is due to a number of factors, both intrinsic and extrinsic, a major contributor is the cell cycle dependence of most conventional chemotherapy drugs. This, the cytotoxic effect of drugs, such as topoisomerase inhibitors and microtubule stabilizing agents, is cell cycle dependent, whereas quiescent cells will be relatively unaffected by these agents [3]. There is a growing recognition of the importance in developing therapies that target both the proliferating and quiescent tumor cell population in order to improve patient outcome [2].

Traditional chemotherapy is usually administered at three-week intervals to facilitate recovery of dividing normal cells, such as those of the bone marrow. It is important to realize that tumor cells, as well as normal cells, may survive treatment and recover between treatment cycles. Such surviving cells have the potential to repopulate the tumor between treatment cycles [2,4]. This problem may not have been given the attention it deserves, and it was only recently shown that chemotherapy leads to reoxygenation and increased proliferation of previously hypoxic and quiescent cells in solid tumors [5]. Tumor repopulation is therefore likely to constitute a major problem in clinical oncology and may be a contributing factor for the limited success of the treatment of patients with advanced malignant disease.

The sequence of how chemotherapeutic drugs are administered will influence their therapeutic effects [6]. Sequence dependence may mainly be due to cell cycle perturbations. Drug interactions should be particularly considered when planning combinations of cytotoxic and cytostatic agents in order not to diminish the efficacy of cell cycle-active chemotherapeutic agents [2]. Cytostatic agents should preferably be administered between courses of chemotherapy to inhibit repopulation, but not given immediately prior to the next round of chemotherapy [2]. Inhibitors of the mTOR/PI3K pathway represent examples of cytostatic agents that have the potential to inhibit tumor repopulation [2,7,8]. A rapamycin analogue was demonstrated to increase the *in vivo* effect of the cytotoxic drug docetaxel when administered between docetaxel treatments to xenografted animals [7]. In a recent study, tumor repopulation was reported to be diminished by treatment with the cyclooxygenase-2 (COX2) inhibitor celecoxib [9].

## 2. Avascular Areas of Solid Tumors Contain Quiescent Cell Populations

**Figure 1:**
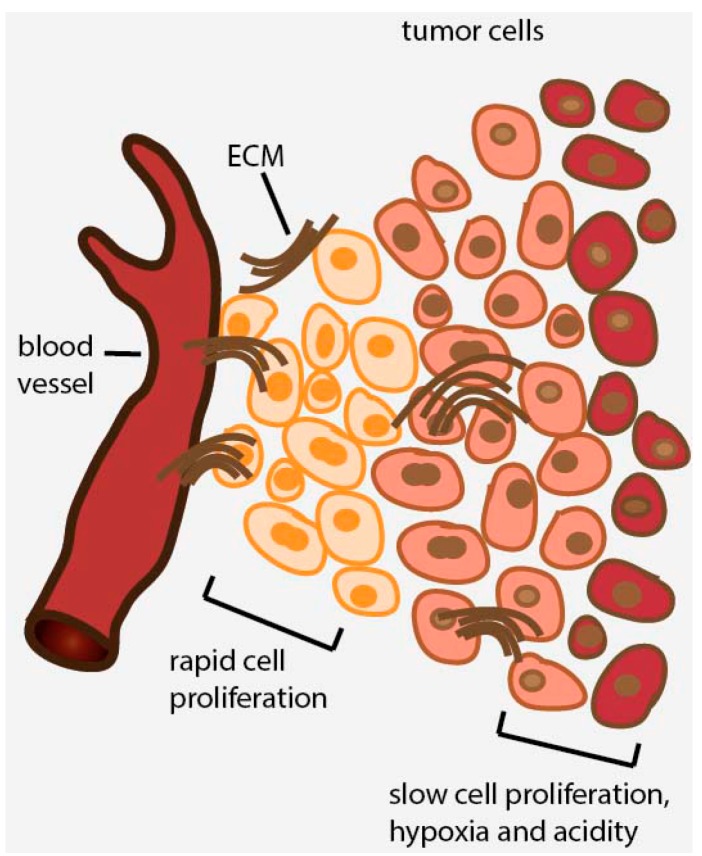
Development of heterogeneity in solid tumors. Continuous outgrowth of the vasculature results in the generation of tumor microenvironments that are characterized by hypoxia and nutrient starvation. Cells in these environments are slowly proliferating and are relatively insensitive to cell cycle-active cancer therapeutics. Extracellular matrix (ECM).

Proliferating tumor cells exist in a fine balance between nutrient supply and demand. Growing tumor cells generally outgrow the supporting vasculature, ultimately leading to cell populations distantly situated (>100 μm) from blood vessels [10]. The vasculature at the tumor site is generally poorly organized [11] with a high degree of compression on the supplying blood vessels, leading to the disrupted flow of nutrients and oxygen to the tumor tissue [12]. In addition, the insufficient perfusion between vessels and tumor tissue, combined with the high production of acidic metabolites, contributes to the development of regions of high acidity within solid tumors [13]. As a consequence, the majority of solid tumors will display regions of increased levels of hypoxia coupled with nutrient starvation and low pH [14] (Figure 1).

Griffin, Kerbel and coworkers [15] coined the term “multicellular resistance” to describe the combination of pharmacokinetic obstacles that limit drug penetrance, as well as cell–cell interactions that lead to altered expression of proteins that are important for cellular sensitivity to anticancer agents. The issue of efficient drug penetrance into tumor tissue, essential to achieve therapeutically-relevant drug concentrations [16,17], is important to consider when developing drugs for solid tumors [18]. Quiescent cells and hypoxic cells show gene signatures that are distinct from cells in well-vascularized areas. Such signatures have been reported to be associated with prognosis and with drug sensitivity [19,20,21].

Considering the clinical problem of regrowth resistance, it would be desirable to identify drugs that show activity on quiescent cells in avascular areas of solid tumors. Such drugs must have a therapeutic window that allows targeting non-proliferating tumor cells while sparing non-proliferating cells in healthy tissues. In addition, such drugs must be able to penetrate into the deep tumor parenchyma.

## 3. Conditional Drug Screening Aimed at Targeting Glucose-Starved Tumor Cells

Cell-based screening for the identification of cytotoxic drugs is usually performed using cell lines grown as monolayers on plastic support. Some culture media contain glucose at concentrations only occurring in severely diabetic individuals, and cells are maintained in atmospheric oxygen levels. The National Cancer Institute has screened >100,000 compounds’ antiproliferative activity on a panel of cancer cell lines (the NCI_60_ cell panel) under such conditions [22]. This has resulted in a wealth of information and the identification of a large number of substances. Most of these drugs are, however, expected to show activity on proliferating tumor cells and may be less effective on slowly-proliferating cells in avascular areas. Unphysiological conditions are not only used during screening; almost all studies of apoptosis induction and cell signaling are performed using tumor cells grown on plastic support in nutrient- and oxygen-rich conditions.

The mean glucose concentration in colon carcinoma tissue is ~2% of the plasma glucose concentration (0.12 *vs.* 5.6 mM) [23]. The cells in solid tumors are therefore in a steady state of nutrition depletion. Deprivation of cultured cancer cell lines of glucose has been reported to lead to resistance to many conventional anticancer agents [24], and it has therefore been of interest to identify agents that inhibit cancer cell viability under conditions of nutrient starvation. A number of drugs have been identified by the group of Esumi and coworkers using this approach. Kigamicin D [24], arctigenin [25], efrapeptin F [26] and pyrvinium pamoate [27] are examples of compounds with preferential antiproliferative activity on tumor cells grown under nutrient-deprived conditions. Kigamicin D is one of the compounds found to be cytotoxic to glucose-starved cancer cells, but not to cells grown in nutrient-rich standard media. Kigamicin D suppressed the in vivo tumor growth of pancreas cancer cell lines in nude mice [24]. Arctigenin is an antitumor antibiotic that displays preferential cytotoxicity under conditions of nutrient starvation and also shows strong in vivo activity against pancreas cancer xenografts [25]. Interestingly, arctigenin was reported to inhibit mitochondrial respiration and to induce a bioenergetic catastrophe [28]. Esumi and coworkers also identified the drug efrapeptin F as preferentially toxic to nutrient-deprived cancer cells [26]. Efrapeptin F has been demonstrated to inhibit the activity of the mitochondrial F1F0-ATPase (complex V) [29].

Pyrvinium pamoate (PP) is an anthelminthic drug (effective against parasitic worms) of particular interest. PP was reported to be extremely toxic to a number of cancer cell lines under conditions of glucose starvation [30]. PP also inhibited the growth of human colon cancer multicellular tumor spheroids (MCTS) and showed antitumor activity *in vivo* [30]. PP inhibited the hypoxic electron transfer chain, the NADH-fumarate reductase system, in mitochondria of tumor cells [27]. Fumarate reductase (FRD) activity has been demonstrated in species such as bacteria and helminths, and evidence that human cancer cells have FRD activity has also been presented [31]. The activity level was reported to be low, but to be increased during culture under hypoxic and glucose-deprived conditions. By inhibiting FRD activity, PP may preferentially target the energy production of cells in hypoxic regions in tumors. PP was also identified by direct screening of MCTS and demonstrated to be a strong inhibitor of oxidative phosphorylation (OXPHOS) [32]. The drug screening findings described here led Esumi and coworkers to address the question of whether nutrient-deprived cancer cells are sensitive to mitochondrial inhibition in general, and this was indeed found to be the case [26].

Activation of the Akt signaling pathway was found to be essential for the ability of cancer cells to survive under low glucose conditions [25,33]. Tolerance to nutrient deprivation was also reported to be associated with the expression of AMP-activated protein kinase (AMPK), an enzyme important for protection from metabolic stress [34]. The screening hits identified by Esumi and coworkers were generally found to block the activation of the Akt pathway [24,25,27].

## 4. Drug Screening Efforts Using Spheroid Models Mimicking the Tumor Microenvironment and Heterogeneity

**Figure 2:**
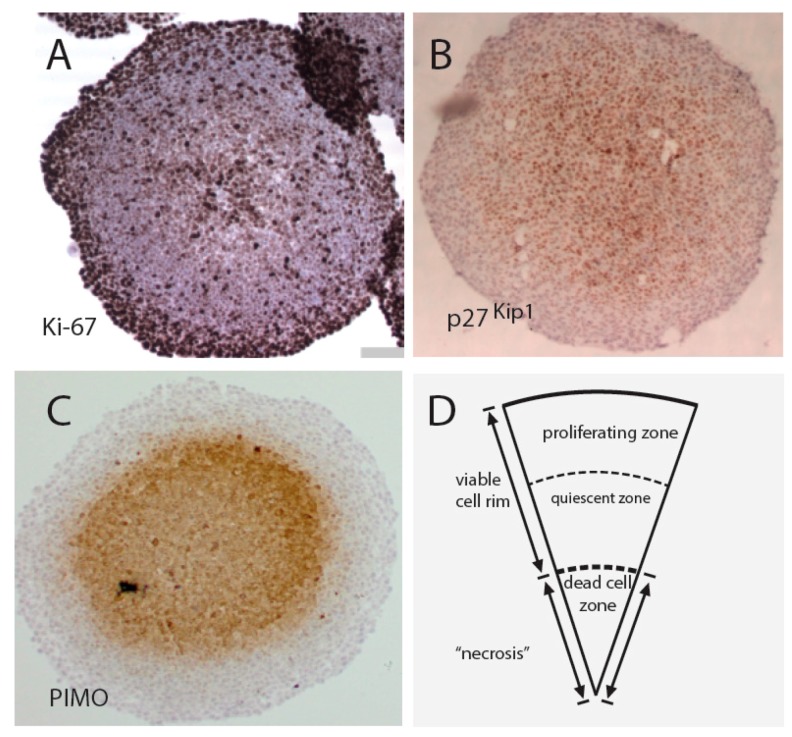
Properties of HCT116 colon cancer spheroids. HCT116 spheroids were grown in 96-well plates for five days, fixed, sectioned and stained with different antibodies. Proliferating cells (Ki-67 positive) are present in peripheral layers (**A**) and quiescent cells (p27^Kip1^ positive) in the core (**B**); Core layers are also severely hypoxic and positive for pimonidazole (PIMO) adducts (**C**); Large spheroids also contain central areas of necrosis (**D**).

The 3D multicellular tumor spheroid model (MCTS) was developed in order to provide a more accurate mimic of the conditions of solid tumors [35,36]. MCTS are heterogeneous with a well-defined geometry, containing proliferating cell populations at surface layers and quiescent cells in the core [36]. HCT116 colon cancer MTCS are shown in Figure 2. In this example, the culture medium contained 25 mM glucose, an unphysiological concentration. The advantage of the use of high glucose concentrations is the development of larger areas of hypoxic cores (staining for pimonidazole adducts), useful for screening purposes (see below). HCT116 MTCS core regions are negative for proliferation marker Ki67, but positive for p27^Kip1^. A possible mechanism for the upregulation of p27^Kip1^ is the downregulation of ERK signaling observed in the MTCS (Figure 2). p27^Kip1^ Protein stability is regulated by a Skp2-dependent mechanism that, in turn, is dependent on MAP (mitogen-activated protein) kinase signaling [37].

Tumor spheroids develop gradients of oxygen, nutrients and catabolites [38]. Oxygen tension values fall steeply in the viable rims of MCTS, suggesting that oxygen consumption rates (OCR) are similar in the inner and outer parts of spheroids [39]. Mitochondrial mass per cell volume has been reported to be constant irrespective of location in MCTS [40,41], whereas mitochondrial function appears reduced in deeper spheroid layers [41]. The core regions of MCTS have lower levels of glucose [42,43] compared to more peripheral layers. The thickness of the viable rim of tumor spheroids is dependent on glucose availability: large necrotic cores are observed in MCTS grown in low-glucose medium [39]. It has been reported that tumor cells grown as monolayers or MCTS show a similar rate of glucose consumption [44,45], suggesting that cells do not increase glucose consumption during hypoxic conditions in MTCS. Lactate levels do, however, increase in large-sized spheroids [46], leading to a more acidic pH in core regions [47]. This finding could be due to limited diffusion of lactate from core regions in MCTS, leading to accumulation.

MCTS are more resistant to chemotherapy compared to monolayer cultures [48,49]. Resistance is not unexpected, considering the presence of quiescent cells (resistant to cell-cycle-active drugs) and the requirement for drug diffusion to reach the core. High drug hydrophobicity (high log *p*) was reported to be required for strong antiproliferative activity in spheroid models [49]. The aspect of hydrophobicity and penetration is not necessarily considered during the development of drugs for solid tumors.

The utilization of MCTS for drug screening is an attractive strategy for anticancer drug development, since a number of relevant factors, such as nutrient starvation, hypoxia and drug penetration, are all accounted for. Traditional methods of MCTS production results in spheroids that are quite heterogeneous in size and not useful for screening [35]. Methods have subsequently been developed where spheroids are produced in microtiter plates, resulting in one similarly-sized spheroid per well [50]. Such MCTS can be used for drug screening campaigns [48,51,52]. Methods using microfluidics-generated double-emulsion droplets for spheroid culture have also been described [53], but whether these can be used for screening is unclear. Different read-outs of cell viability and apoptosis can be used for screening, including acid phosphatase [54], green fluorescent protein expression [32], methylene blue [55], caspase-cleaved keratin 18 [51] and high-content analysis using fluorescent dyes [56].

A colon MCTS screen using 10,000 compounds from ChemBridge (San Diego, CA, USA) resulted in the identification of VLX600, a drug that induces preferential cell death of tumor cells in spheroid cores and has *in vivo* activity [57]. The compound induces AMPK phosphorylation in tumor cell lines, but not in immortalized cells. VLX600 was found to reduce mitochondrial OXPHOS, particularly the rate of uncoupled respiration. This effect was associated with a strong decrease in MCTS hypoxia, measured as pimonidazole-stained fraction [57], and also reduced hypoxia in colon carcinoma xenografts *in vivo* (Fryknäs *et al.*, unpublished data). As might be expected, exposure to VLX600 sensitized colon cancer cells to glucose starvation. The precise molecular mechanism of action of VLX600 has been elucidated using gene expression profiling and shown to be iron chelation (Fryknäs *et al.*, unpublished data). The activity of the compound on quiescent tumor cells was hypothesized to be due to these cells showing a decreased metabolic plasticity (*i.e.*, limited ability to switch between different modes of energy production) [57].Two other screens of MCTS have been described using smaller drug libraries consisting of bioactive molecules. Wentzel *et al.* [56] screened two drug libraries, one consisting of 640 FDA-approved drugs and another containing 480 drugs with known mechanisms of action. In a separate study, 1600 compounds with documented clinical history were screened using MCTS [32]. The use of chemical libraries containing drugs with known modes of action has the advantage of giving an immediate recognition of the mechanisms of cytotoxic activity. Compounds that affect mitochondrial function were identified in both of these screens. All hits from Wentzel *et al.* [56] interfered with the proper function of the respiratory chain, either by acting as inhibitors or uncouplers of the respiratory chain. Thus, the exact target in the respiratory chain seems to be irrelevant, as complex I inhibitors induce similar phenotypes as complex III/V inhibitors or uncouplers of the respiratory chain. The authors interpreted their findings to suggest that low levels of glucose in MCTS core regions do not allow glycolysis to provide sufficient ATP (adenosine triphosphate), resulting in cells with dependence on respiration for survival. Supplementation of glucose was indeed found to decrease the toxicity of the screening hits. These authors also showed that the combination of mitochondrial inhibitors and conventional cytostatic drugs led to increased levels of cell death in MCTS [56]. The study by Senkowski and coworkers [32] led to the identification of five compounds with selective activity on MCTS: closantel, nitazoxanide, niclosamide, pyrvinium pamoate and salinomycin. These compounds have all been described to target mitochondrial function by different mechanisms. Niclosamide, closantel and nitazoxanide share an identical pharmacophore and have been demonstrated to possess uncoupling activity [58,59,60]. Pyrvinium pamoate also inhibits the mitochondrial OXPHOS and was discussed above. The identification of salinomycin by MCTS screening is interesting considering that the same drug was identified in a screen for agents active on cancer stem cells [61]. Salinomycin has also been described to inhibit OXPHOS [62].

## 5. Drug Screening Using Cancer Stem Cells

Cancer stem cells (CSC) have attracted an enormous interest from the scientific community during recent years. According to the cancer stem cell model, a limited subset of cancer cells has the capacity to propagate tumors based on their capacity for self-renewal and their ability to generate differentiated progeny [63]. CSCs have been reported to display increased resistance to conventional chemotherapeutic agents and to radiation [64,65] and are therefore likely candidates for therapy-resistant cells that give rise to tumor recurrences. In a recent study, tumor repopulation between chemotherapy cycles was shown to be due to stimulation of CSC proliferation by prostaglandin E2 (PGE-2) release by neighboring cells [9]. Whether CSCs survive chemotherapy and repopulate tumors is, however, somewhat controversial [66]. Similar to quiescent cells in spheroid cores, CSC are thought to reside in a hypoxic niche [67]. It remains possible that the position of tumor cells in relation to the vasculature, rather than stemness, determines the susceptibility to therapy and the potential for recurrence.

A number of groups have used CSC in drug screening campaigns (reviewed in [68]). Gupta *et al.* [61] performed a drug screen for compounds that would display selective toxicity for breast CSCs. This endeavor resulted in the identification of the antibacterial drug salinomycin [61]. The authors reported that salinomycin inhibits mammary tumor growth *in vivo* and that the drug induced alterations in gene expression, indicating a loss of the CSC phenotype. Salinomycin is an OXPHOS inhibitor [32,62] and has also been identified by MCTS screening [32]. The therapeutic window of salinomycin is limited, and it is presently unclear whether this drug can be developed for clinical applications [69]. Ovarian cancer and breast cancer stem-like cells have also been used for drug screening, resulting in the identification of niclosamide [70,71], a mitochondrial uncoupler [59]. Niclosamide also has activity on colon cancer MCTS [32]. Sztiller-Sikorska utilized a library of natural products to screen for drugs with activity against melanoma cells with self-renewing capability [72]. One of the compounds identified was streptonigrin, shown to decrease the respiration of isolated rat liver mitochondria [73].

Metformin is believed to be the most commonly-prescribed anti-diabetic drug in the world and functions by increasing cellular glucose uptake [74]. Metformin inhibits mitochondrial OXPHOS [75,76], but this effect is controversial, since it occurs at high drug concentrations and may be physiologically irrelevant [77]. Metformin also inhibits 5′-adenosine monophosphate (AMP)-activated kinase (AMPK) [78] and was recently shown to be an inhibitor of mitochondrial glycerophosphate dehydrogenase (mGPD) activity [79]. Metformin was reported to selectively induce apoptosis of pancreatic CSC, by a mechanism involving a bioenergetic catastrophe associated with ROS induction and reduced mitochondrial transmembrane potential [80]. The frequency of tumor incidence in diabetic patients is decreased by metformin use [81], and metformin shows anti-neoplastic activity in mouse animal models [82,83]. For a recent comprehensive review on metformin, see [77].

## 6. Tumor Cell Metabolism Is Dependent on Oxidative Phosphorylation (OXPHOS)

Increased glycolysis under aerobic conditions (“Warburg effect”) is observed in most cancer cells [84]. This effect is currently believed to be associated with the increased rates of proliferation of tumor cells where glycolysis contributes the building blocks required for anabolic processes [85]. Upregulation of glycolysis is also necessary for the synthesis of reducing equivalents (NADPH) via the pentose phosphate pathway [86]. The Warburg effect may be explained by the increased expression of the M2-form of pyruvate kinase observed in rapidly-proliferating cells [87]. The observations of elevated glycolysis in tumor cells have led to efforts aimed at targeting this process. The glucose analogue 2-deoxy-d-glucose (2-DG) is transported into cells and phosphorylated by hexokinase. The phosphorylated form of 2-DG cannot be metabolized further, leading to its accumulation and inhibition of glycolysis. 3-bromopyruvate (3-BP) is a potent inhibitor of glycolysis and an alkylating agent. 3-BP has been demonstrated to inhibit hexokinase-II activity *in vitro* [88]. A number of other targets have also been described, and the effects of 3-BP appear to be mediated mainly by its alkylating properties, notably towards thiols [89]. Dichloroacetate (DCA) is an inhibitor of pyruvate dehydrogenase kinase, a mitochondrial enzyme pyruvate dehydrogenase that converts pyruvate to acetyl CoA (Coenzyme A). Exposure leads to the induction of a switch from aerobic glycolysis to glucose oxidation, subsequently leading to a decreased mitochondrial membrane potential and sensitization to apoptosis [90]. A phase I study for DCA has been initiated [91]. Cancer cell metabolism is emerging as a major arena for the development of cancer therapeutics; this review is not intended to provide a comprehensive overview of this area of research (see [92,93,94]).

Although Warburg hypothesized that mitochondrial bioenergetics is defective in tumor cells [95], it has become increasingly clear that mitochondria in fact are essential for the proliferation of cancer cells [96,97]. Studies of the energy budgets of various cancer cells under normoxic conditions concluded that most of the ATP in cancer cells in fact emanates from mitochondria. Zu and Guppy reported that in a panel of 31 tumor cell lines, the average contribution of glycolysis was 17%, compared to 20% in normal cells [98]. These results are supported by later studies: mitochondrial respiration indeed continues to operate normally at rates proportional to oxygen supply [86]. Mandujano-Tinoco and coworkers reported that OXPHOS was the predominant source of ATP both in quiescent and proliferating cell layers in MTCS (93% of total ATP was derived from OXPHOS in quiescent cells, compared to 98% in proliferating cells) [99]. Downregulation of OXPHOS in energy-demanding tumors cells is, in fact, counterintuitive considering the inefficiency of glycolysis as a means of ATP production. Mitochondrial metabolism is considered to be important for tumor growth, both by providing energy in the form of ATP, as well as various metabolic intermediates necessary for anabolic reactions [100]. Direct evidence for the dependence of tumor cells on mitochondria comes from the study of ρ^0^ tumor cells, where mtDNA has been eliminated through growth in ethidium bromide. ρ^0^ Tumor cells display reduced growth rates and a decreased tumorigenicity *in vivo* [101,102,103]. Furthermore, overexpression of TFAM (mitochondrial transcription factor A) leads to increased mitochondrial biomass and stimulates cell proliferation of cancer cells [104]. Two known regulators of mitochondrial metabolism, PGC-1α (peroxisome proliferator-activated receptor gamma coactivator 1-α) and ERRα (estrogen-related receptor α), have also been reported to stimulate the proliferation various forms of carcinomas and of melanomas [105].

The commonly-used deduction that energy production under hypoxic conditions is met by increased glycolysis does not account for the fact that also glucose levels are limited in hypovascular tumors [23,106,107]. Other sources of energy production are available and have been proposed to be used by cells in hypovascular areas. The reductive metabolism of glutamine-derived α-ketoglutarate to be used for the synthesis of acetyl-CoA is one such pathway [108]. Increased utilization of glutamine for energy generation has also been demonstrated in cells exposed to acidic conditions, occurring in the deep tumor parenchyma. Energy generation will in this instance require oxygen and mitochondrial function, since glutamine supports mitochondrial cell respiration through the TCA (tricarboxylic acid) cycle [109].

Some evidence points to increased OXPHOS activity in human tumors. Histochemical staining of the *in situ* enzymatic activity of cytochrome C oxidase (COX; complex IV) showed abundant activity in tumor cells compared to adjacent stromal cells and normal epithelial cells [110]. Similar results were observed for complex I and II activities [110]. Additional studies have shown that the expression of components involved in mitochondrial biogenesis (nuclear respiratory factor 1 (NRF1), mitochondrial transcription factor A (TFAM) and mitochondrial transcription factor B1 (TFB1M)), mitochondrial translation and mitochondrial lipid biosynthesis (Golgi phosphoprotein 3 (GOLPH3) and GOLPH3L) are upregulated in human breast carcinoma cells and downregulated in adjacent stromal cells [111]. Furthermore, the elevated expression of the mitochondrial markers TIMM17A and TOMM34 is associated with poor clinical outcome [112,113,114].

## 7. Mitochondrial OXPHOS as a Therapeutic Opportunity to Target Non-Proliferating Tumor Cells

Mitochondrial inhibitors have been known to possess anti-tumor activity for many years [26,115,116]. Examples of such compounds are the dye Rho123 [115], which inhibits OXPHOS [117], the complex V inhibitor efrapeptin F [26] and mitochondria-targeted lipophilic cations [118]. More recently, screening of a library of FDA-approved drugs for drugs with tumor cell selectivity led to the identification of the antimicrobial drug tigecycline. This drug functions as an inhibitor of mitochondrial translation [119,120].

We conclude that the drug screening efforts using MCTS, glucose-starved cells and CSC reviewed in this article have frequently resulted in the identification of mitochondrial inhibitors. In some reports, all screening hits were found to have effects on OXPHOS. When interpreting results from drug screens, it is important to keep in mind that different targets may show differences in “druggability”. Thus, mitochondrial energy metabolism, which is dependent on a large number of components and intact membrane structures, may be more sensitive to drug intervention than other potential targets. It is nevertheless interesting that mitochondrial inhibitors are encountered in screens performed using different cell lines, cancer stem cells, different culture conditions (glucose-starved monolayers, MCTS) and different platforms (screening libraries, read-outs, *etc.*). This has led us and others to hypothesize that energy production in tumor cells situated in the deep tumor parenchyma is vulnerable and cannot tolerate even limited decreases in OXPHOS [56,57].
